# Tracers and Imaging of Fatty Acid and Energy Metabolism of Human Adipose Tissues

**DOI:** 10.1152/physiol.00012.2023

**Published:** 2023-12-19

**Authors:** André C. Carpentier

**Affiliations:** Department of Medicine, Division of Endocrinology, Centre de Recherche du Centre Hospitalier Universitaire de Sherbrooke, Université de Sherbrooke, Sherbrooke, Quebec, Canada

**Keywords:** adipose tissue, brown adipose tissue, cardiometabolic diseases, positron emission tomography, tracer methods

## Abstract

White adipose tissue and brown adipose tissue (WAT and BAT) regulate fatty acid metabolism and control lipid fluxes to other organs. Dysfunction of these key metabolic processes contributes to organ insulin resistance and inflammation leading to chronic diseases such as type 2 diabetes, metabolic dysfunction-associated steatohepatitis, and cardiovascular diseases. Metabolic tracers combined with molecular imaging methods are powerful tools for the investigation of these pathogenic mechanisms. Herein, I review some of the positron emission tomography and magnetic resonance imaging methods combined with stable isotopic metabolic tracers to investigate fatty acid and energy metabolism, focusing on human WAT and BAT metabolism. I will discuss the complementary strengths offered by these methods for human investigations and current gaps in the field.

## Introduction

White adipose tissue and brown adipose tissues (WAT and BAT) regulate long-chain fatty acid exposure of lean organs in the postabsorptive and postprandial states through fatty acid storage, mobilization, and oxidation. These processes are of high interest in cardiometabolic disorders because of the strong evidence for the early implication of increased systemic lipid fluxes, ectopic fatty acid deposition and lipotoxicity in the development of cardiovascular complications (1), insulin resistance, and beta-cell (2), cardiac (3), kidney (4), and liver damage (5, 6). Metabolic tracers with invasive (e.g., blood samples, biopsies) and/or noninvasive (e.g., molecular imaging) sampling methods are essential tools for the investigation of adipose tissue fatty acid and energy metabolism and for the quantification of lean organ fatty acid fluxes.

The use of tracers for the study of metabolism was introduced in the 1930s by Shoenheimer and Rittenberg in a series of early investigations (7, 8). These investigators initially used ^2^H_2_O to label fatty acids and other organic molecules fed to laboratory animals. The fractional deuterium labeling rate in various biological samples from these animals was then determined using fastidious methods exploiting the differential physical properties of the diluted heavy water (change in density or refractive index) obtained after combustion and a series of freezing and distillation purification steps. These pioneering studies also initially used ^15^N-labeled amino acids and [^13^C]acetate. The tracer-based methods of quantification of in vivo systemic energy metabolic fluxes, such as glucose and fatty acids, have thereafter relied on radioisotopes (mainly ^14^C- and ^3^H-labeled tracers) during decades before stable isotopes (mainly ^2^H- and ^13^C-labeled tracers) were reintroduced and largely adopted more recently after improvements and broader availability of mass spectrometry techniques (9, 10). In 2024, tracer methods remain the state-of-the-art approaches to define and quantify critical in vivo pathophysiological processes such as insulin resistance and endogenous glucose production, and hundreds of studies are published every year using these methods. These systemic tracer-based protocols have recently been extended and refined by the addition of molecular imaging techniques such as positron emission tomography (PET) to investigate insulin sensitivity and glucose fluxes at the organ and tissue levels in vivo (11, 12). Nowadays, in vivo tracer-based methods for the quantification of in vivo energy metabolic fluxes at the systemic and organ-specific levels are essential to understand the pathophysiology and pathogenesis of highly morbid and prevalent multisystemic diseases including obesity, type 2 diabetes (T2D), metabolic dysfunction-associated steatohepatitis [MASH; formerly nonalcoholic steatohepatitis (13)], cardiovascular events and strokes, and cardiac and kidney failure. The classical tracer-based methods, often complemented by PET and/or MRI, are offering powerful investigation tools that will continue to unravel new knowledge in the foreseeable future.

This brief review will summarize WAT and BAT fatty acid and energy metabolism and the regulation of postprandial fatty acid fluxes and will discuss methods to investigate these key pathophysiological processes. The discussion will especially focus on PET methods that we and others have introduced to investigate fatty acid and WAT and BAT energy metabolism in humans and describe their advantages and limitations compared to conventional tracer methods and MRI.

## Overview of Postprandial Fatty Acid Metabolism in Humans

**FIGURE 1.**
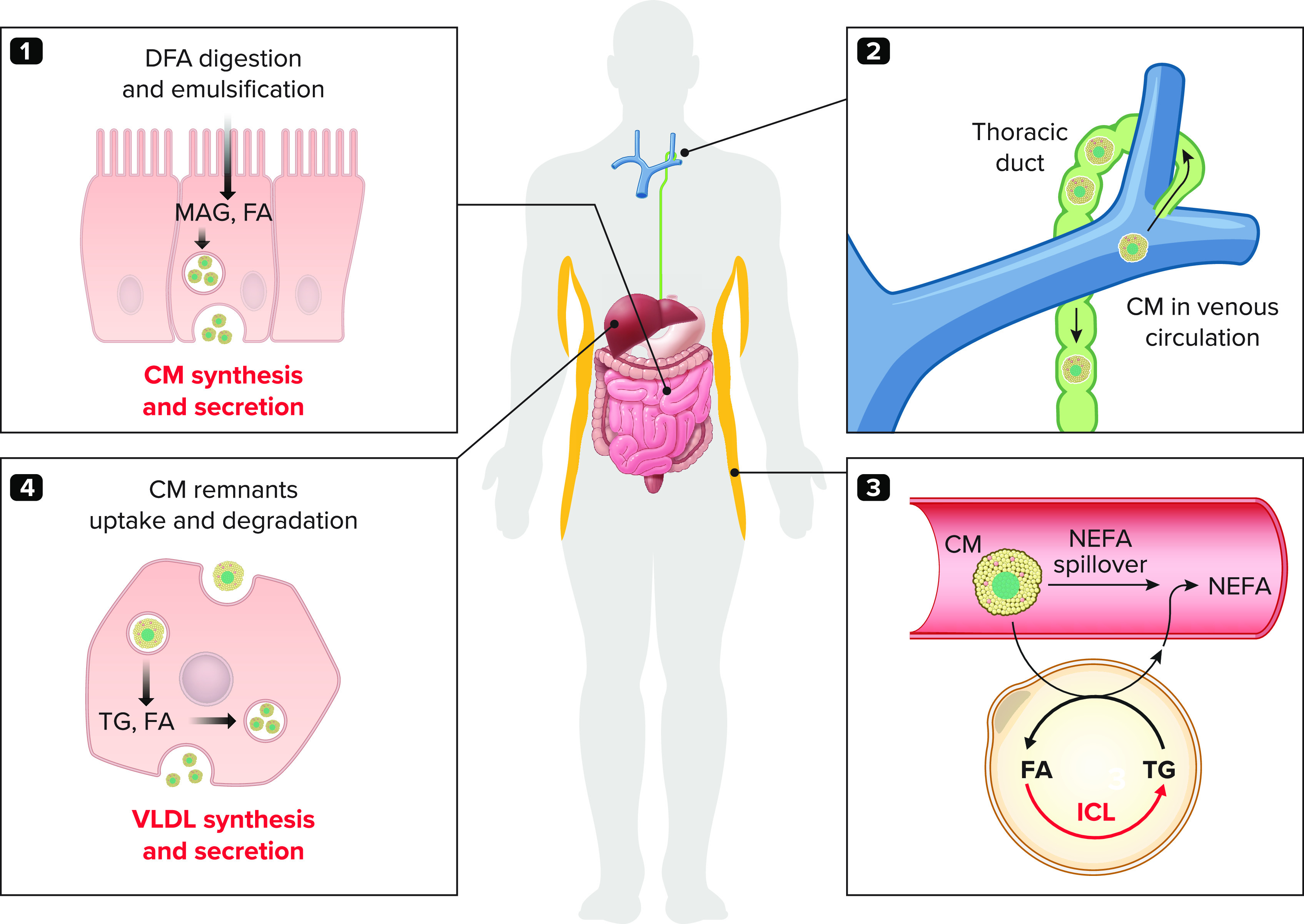
**Postprandial fatty acid metabolism** Postprandial fatty acid metabolism from dietary fatty acid digestion and secretion into chylomicrons (*1*), to distribution of chylomicrons in the venous circulation via the thoracic duct (*2*), lipoprotein lipase-mediated intravascular lipolysis of chylomicron-triglycerides and nonesterified fatty acid spillover in adipose tissues (*3*), and chylomicron remnants uptake and very-low density lipoprotein secretion by the liver (*4*). Metabolic steps that are increased in prediabetes and type 2 diabetes are depicted in red. CM, chylomicron; DFA, dietary fatty acids; FA, fatty acids; ICL, intracellular lipolysis; LPL, lipoprotein lipase; MAG, monoacylglycerol; NEFA, nonesterified fatty acids; TG, triglycerides; VLDL, very-low-density lipoprotein.

The postprandial state can be functionally defined, as opposed to the postabsorptive (or fasting) state, as the period during which net positive systemic flux of a given meal substrate occurs. This functionally defined postprandial period differs between nutrients. It is shorter (i.e., less than 5 hours) for metabolites such as glucose and amino acids that are efficiently absorbed by the intestine, rapidly distributed through the portal vein, and rapidly taken up by the liver and other organs. In contrast, it is much longer for long-chain fatty acids (i.e., more than 6 hours) that require several relatively slow processing steps (FIGURE 1): *1*) digestion through dietary triglyceride (TG) lipolysis, absorption through intestinal uptake, reesterification into TG, and reassembly into chylomicrons within the enterocytes; *2*) secretion into the intestinal lymph and distribution through the thoracic duct and then in the systemic venous circulation up to the tissue microcirculation; *3*) lipoprotein lipase (LPL)-mediated lipolysis of chylomicron-TG at the endothelial luminal side to produce nonesterified fatty acids (NEFA) that are taken up into tissues; and *4*) chylomicron remnants uptake by the liver with recycling of some of the dietary fatty acids into very-low-density lipoprotein (VLDL)-TG secreted back into the circulation. The postprandial period of dietary long-chain fatty acids (DFA) is further prolonged by recirculation of these fatty acids into the plasma NEFA pool from adipose tissue release, a process coined “DFA spillover” (14). In addition, a significant fraction of DFA can be retained in the intestinal epithelium as chylomicrons, which will be secreted with subsequent oral carbohydrate intake (15–17). Therefore, the postprandial fatty acid period of a given meal intake is actually bi- or multiphasic. The postprandial period for DFA is also affected by many pathophysiological factors such as meal composition and size, time of day, type of fat ingested (18–21), and the presence of disorders of the exocrine pancreas, intestine, autonomic nervous system, or lipid metabolism such as LPL deficiency or dysfunction (22).

In prediabetes and T2D, postprandial hyperlipidemia (i.e., increased circulating levels of chylomicrons, VLDLs, and their remnants) occurs because of a combination of impaired LPL-mediated clearance and increased intestinal and hepatic production rates of TG-rich lipoproteins (1, 23). Intestinal chylomicron-TG assembly and secretion are downregulated by insulin, gut hormones, and neural signals and upregulated by dietary and systemic glucose and fatty acid availability (23, 24). Hepatic VLDL-TG secretion is also downregulated by neural and hormonal signals and upregulated by nutrient availability, including fatty acid fluxes to the liver from TG-rich lipoprotein remnants and plasma NEFA, and dietary carbohydrates (through upregulation of hepatic de novo lipogenesis) (23, 25, 26). Along with the intestine and liver, WAT (adipocytes and the stroma-vascular cells) is the most important regulator of postprandial fatty acid metabolism (FIGURE 1). WAT is an important contributor of chylomicron-TG and VLDL-TG peripheral LPL-mediated lipolysis and clearance and therefore of the production of TG-rich lipoprotein remnants (1). In turn, these remnants contribute to hepatic fatty acid flux and the modulation of hepatic metabolism, including VLDL-TG secretion. Furthermore, WAT is the main regulatory hub for the plasma NEFA pool that is also increased in prediabetes and T2D (14). Two processes largely occurring in WAT contribute to this pool: *1*) intracellular TG lipolysis; and *2*) the aforementioned DFA spillover, that is, the release of NEFA from the LPL-mediated lipolysis of chylomicron-TG and, to a small extent, VLDL-TG (27–29). These WAT-derived NEFA fluxes have been shown to contribute ∼90% and >70% of VLDL-fatty acids at fasting and in the postprandial state in healthy subjects, respectively (30). Although the contribution of de novo lipogenesis is increased in subjects living with metabolic dysfunction-associated steatotic liver disease [MASLD (13)], WAT-derived NEFA fluxes still account for the bulk of VLDL-fatty acids in the postprandial state (i.e., up to 74%) (31). Higher WAT NEFA flux is associated with greater liver TG content and a dysmetabolic profile (32), and resistance to insulin-mediated suppression of WAT NEFA flux is closely associated with hepatic TG content (6) and inflammation and fibrosis (33). WAT-derived NEFAs are also major contributors to key pathogenic processes, such as increased hepatic VLDL particle secretion and glucose production (23, 34). Hence, fatty acid fluxes are key pathogenic mechanisms regulated by WAT that need to be measured in humans not only at the systemic but also at the organ and tissue levels, using methods that will be discussed in Investigation of Postprandial Fatty Acid Metabolism Using Combination of Stable Isotope Tracers and Metabolic Imaging. The very high utilization of circulating fatty acids by cold-activated BAT in rodents (35) also justifies special attention to fatty acid and energy metabolism in these adipose tissue depots in humans, with methods that will be discussed in Investigation of BAT Metabolism in Humans Using Combination of Stable Isotope Tracers and Positron Emission Tomography.

## Investigation of Postprandial Fatty Acid Metabolism Using Combination of Stable Isotope Tracers and Metabolic Imaging

Table 1 details the main characteristics, advantages, and limitations of classic stable or radioactive isotope tracer and metabolic imaging methods to investigate WAT and BAT fatty acid and energy metabolism. Many groups have established the use of a combination of intravenously and oral administration of classic stable or radioactive isotope-labeled fatty acids (i.e., ^13^C-, ^14^C-, ^2^H-, and/or ^3^H-labeled long-chain fatty acids) (18, 19, 36–39). This approach is the gold standard for quantifying systemic DFA spillover and NEFA fluxes. One major advantage of stable isotopes is the capacity to measure downstream metabolic partitioning with a combination of multiple tracers [e.g., ketogenic and citrate synthase fluxes in the liver (40)]. In combination with breath ^13^CO_2_ enrichment determination and indirect calorimetry, these methods also provide estimations of dietary and/or circulating fatty acid systemic oxidation and nonoxidative metabolic rates (41, 42). These measurements have also been combined with arteriovenous sampling or biopsy techniques to estimate WAT, muscle, cardiac, and even hepatic fatty acid uptake and release (31, 43, 44). Measurement of stable isotopic tracer incorporation in adipose tissue lipids has been limited by the sensitivity of mass spectrometry approaches at low circulating tracer concentrations. Tritiated fatty acids have been used to measure adipose tissue fatty acid storage in humans (45, 46). Spontaneously occurring adipose tissue labeling with environmental ^14^C from nuclear testing fallouts has even been exploited to measure long-term lipogenesis and adipocyte turnover from human adipose tissue biopsies (47, 48). This quantification of WAT tracer enrichments using conventional radioactive or stable isotopic tracers does not require in situ measurement and can be performed remotely and at a later time, using largely available mass spectrometry or radioactivity spectrometer facilities and expertise. However, these methods are invasive as they require biopsy and/or catheterization of selective vascular beds that are generally limited to one organ or anatomic region (e.g., abdominal WAT, leg, or forearm), are prone to sampling dilution errors, are very labor intense and costly, and have a very limited time resolution due to the slow sampling process (i.e., cannot measure very fast changes in tracer concentrations and cannot continuously track metabolic processes at the tissue level).

**TABLE 1.** Relative advantages and limitations of tracer and molecular imaging methods for fatty acid and energy metabolism

Description	Advantages	Limitations
Conventional radioactive or stable isotope tracers	Measure of systemic metabolic fluxes Combined with breath ^13^CO_2_ enrichment to estimate systemic oxidation rates Combined arteriovenous sampling and/or tissue biopsy to measure local tissue fatty acid uptake, secretion, and esterification Delayed and remote sample measurement feasible with relative availability of mass spectrometry platforms and expertise	Sampling dilution and errors Limited time resolution Invasive and not amenable to simultaneous measurements in most tissues
Magnetic resonance imaging/spectroscopy	Gold standard for noninvasive measurement of tissue fat composition Excellent spatial and time resolution Can be used with stable isotopic tracers to measure tissue fatty acid esterification into intracellular triglycerides	Low molecular sensitivity severely limiting the capacity to detect small changes in tissue lipid composition and stable isotope tracer enrichment Limited availability High cost
Positron emission tomography	Most sensitive noninvasive molecular technique High versatility of metabolic tracers High time resolution Simultaneous scanning of multiple organs possible	Ionizing radiations Necessitate a cyclotron and radiopharmacy facilities and expertise on-site for the production of many tracers Generic signal preventing simultaneous use of PET tracers Low spatial resolution and tissue spillover of signal High cost

Simultaneous measurement of fatty acid uptake and metabolism in all internal organs requires noninvasive methods able to sample multiple tissues continuously over time. Magnetic resonance spectroscopy (MRS) and imaging (MRI) have been applied to quantify metabolic processes at the tissue level. MRI and MRS are very safe and free of exposure to ionizing radiations. These techniques display very good spatial and time resolutions and are state-of-the-art in measuring tissue composition (i.e., water and fat content, lipid composition, etc.). A major strength of these methods is the capacity to quantify selectively the relative concentration of multiple pools of lipids (e.g., TG saturated and unsaturated fatty acids) (49, 50). Specific quantification of in vivo stable isotopic enrichment of tissue TG content (e.g., ^2^H, ^13^C) is feasible (51–53) but currently limited by the very low molecular sensitivity of MRI and MRS (in the order of 10^−3^ M) (54). This low sensitivity makes currently impossible the tracking of rapid changes in tissue tracer concentrations, limiting the potential applications to relatively large changes of highly concentrated metabolic tissue pools of substrates (e.g., change in liver TG content after meals or exercise training; reduction of BAT fat fraction during acute cold exposure or after bariatric surgery) (40, 55–59). These methods are also relatively unavailable for research and are costly.

PET is a powerful technique to measure energy substrate uptake and metabolism at the tissue level. First, positron-emitting isotopes such as ^11^C, ^18^F, ^15^O, or ^13^N can be used to label common natural energy substrates or chemically modified analogs that confer desirable metabolic properties to target specific metabolic processes. This makes PET a highly versatile modality limited only by the expertise and resources of the local radiopharmacy unit. Second, PET is by far the most sensitive imaging modality, being able to detect tracer concentrations in the range of 10^−15^ M (54). This very high sensitivity allows the intravenous administration of very small doses of tracer analogs that would otherwise affect metabolic processes (e.g., ^18^F-fluoro-deoxiglucose at high doses blocks glycolysis). PET tracers thus respect an essential characteristic of ideal tracers, i.e., very low tracer-to-tracee concentration ratios, which is not always achievable with stable isotopic tracers. Third, PET has a very high and versatile time resolution able to detect changes in signal within a few seconds and also to integrate slower fluctuations of signal over minutes to hours. Thus very fast and very slow kinetic processes can be measured by this imaging modality. PET also allows simultaneous scanning of multiple organs/anatomic structures. The main limitations of PET are *1*) the exposure to ionizing radiations; *2*) the limited half-life of many metabolic tracers (e.g., 19 min for ^11^C) forcing on-site production and reducing the widespread use of some tracers; *3*) the detection of a generic signal (i.e., gamma-radiation) that makes indistinguishable the signal of different positron-emitting tracers and renders impossible simultaneous acquisition with multiple PET tracers; *4*) the limited spatial resolution and spillover of the tracer signal making impossible selective imaging of small anatomical structures very close to metabolically active organs (e.g., pericardial fat); and *5*) the high cost of this method. PET is also minimally invasive as it requires intravenous administration of the tracer, although oral administration can be performed in some instances (see below). PET scanners and the tracer ^18^FDG are widely available in academic centers, although time for research is usually very limited.

Ingested DFAs are delivered over at least 4–6 hours to organs, a rate that is orders of magnitude slower than the fatty acid oxidation rate in tissues. Therefore, tracers of natural long-chain fatty acids cannot measure DFA uptake in tissues that oxidize fatty acids extensively, such as the heart, liver, and BAT, eliminating most of the signal almost as soon as the tracer is taken up in these tissues. To address this issue, we developed and validated orally administered [^18^F]fluoro-thia-heptadecanoic acid ([^18^F]FTHA) PET as a useful method for DFA flux analysis (42, 60–68). [^18^F]FTHA is a long-chain fatty acid analog that accumulates in oxidative and nonoxidative cellular pathways, owing to its thiol group in the acyl chain that impairs its beta-oxidation (69). This property allows the use of linearization methods to measure the relatively slow incremental uptake over time of DFA in various organs, including those with very efficient fatty acid oxidative metabolism such as the heart and liver. This property also allows whole body (“static”) PET scanning for the determination of relative tracer partitioning in most organs. The rate of [^18^F]FTHA fractional tissue extraction is similar to that of [^11^C]palmitate in vivo, making this tracer reliable for quantifying long-chain fatty acid uptake rate in organs (70, 71). We found that [^18^F]FTHA given orally first reaches the systemic circulation as chylomicron-TG (61). Over time, [^18^F]FTHA in chylomicron-TG then undergoes recycling into the NEFA pool through DFA spillover from WAT. Orally administered [^18^F]FTHA therefore measures DFA that reaches organs both as chylomicron-TG and through DFA spillover from WAT, thus quantifying the total contribution of DFA to organ fatty acid exposure. The orally administered [^18^F]FTHA PET measurement assumes that [^18^F]FTHA is hydrolyzed from chylomicron-TG similarly to other long-chain fatty acids in the microcirculation of organs. One limitation of this method is the incapacity to directly assess DFA partition in many visceral fat depots due to the important intestinal radioactivity spillover in surrounding tissue images. Intra-abdominal adipose tissue DFA partitioning is thus extrapolated from perirenal adipose tissue DFA uptake.

The orally administered [^18^F]FTHA PET method allowed us to track DFA uptake over 6 hours after meals in most organs of the body, especially WAT depots, skeletal muscles, the liver, and the heart), that cannot be obtained from other usable fatty acid metabolic tracers in vivo (Table 2). It led to novel published observations about DFA metabolism in humans. First, compared to healthy lean subjects, subjects living with prediabetes and abdominal obesity display increased cardiac DFA partitioning associated with subclinical ventricular dysfunction (62), which is reversible with modest weight loss induced by a change in lifestyle (63). In contrast, 7-day caloric restriction in subjects living with prediabetes leads to a further increase in cardiac DFA partitioning despite a small reduction in weight and improvement in insulin sensitivity (65). In healthy subjects, 2 weeks of calorie overfeeding leading to reduced insulin sensitivity without significant change in body composition also leads to an 8% increase in DFA WAT partitioning with reciprocal reduction in skeletal muscle and cardiac DFA partitioning (66). We also found that intra-abdominal WAT DFA uptake (extrapolated from perirenal fat) was increased within 2 weeks after bariatric surgery in subjects with T2D, associated with improved liver insulin sensitivity and reduced cardiac DFA partitioning (42).

**TABLE 2.** Relative advantages and limitations of some fatty acid tracers usable in humans

Description	Advantages	Limitations
^3^H-labeled fatty acids	Advantages of conventional tracers (see Table 1) Low cost, high availability of spectrophotometry for ex vivo signal measurement in plasma or tissues High sensitivity allowing quantification in tissue samples Low radioactivity exposure	Limitations of conventional tracers (see Table 1) Cannot be used for imaging Generic signal that necessitates the separation of metabolites for specific determination of metabolic pathways
^2^H- and ^13^C-labeled fatty acids	Advantages of conventional tracers (see Table 1) Large array of tracers available for different fatty acids Chromatography/mass spectrometry methods largely available allowing measurement of tracer enrichment of specific metabolite pools	Limitations of conventional tracers (see Table 1) Limited sensitivity relative to radioactive tracers for study of tissue metabolic rates Limited sensitivity for in vivo imaging using MRS methods
[^11^C]palmitate	Advantages of PET (see Table 1) Allows tissue-specific determination of palmitate uptake, and oxidative and nonoxidative metabolism.	Limitations of PET (see Table 1) Requires dynamic PET scanning and multicompartmental modeling with correction for circulating ^11^CO_2_ Measurement is limited to organs in the field of view of the scanner
[^18^F]FTHA	Advantages of PET (see Table 1) Trapped in mitochondria during its oxidation, allowing the quantification of relatively slow metabolic processes (such as dietary fatty acid partitioning in different organs, when administered orally) Amenable to whole body (“static”) imaging that determines the relative tissue partitioning of the tracer	Limitations of PET (see Table 1) Cannot determine tissue specific metabolism, only uptake Measurement in some tissues (e.g., some intra-abdominal fat depots) is limited by intestinal radioactivity spillover

By its very nature and purpose, orally administered [^18^F]FTHA PET does not allow the quantification of oxidative and nonoxidative fatty acid metabolic rates in the tissues. Furthermore, it quantifies tissue DFA uptake but not total NEFA uptake, which is a very important pool of fatty acids available to tissues in the postprandial state. We have therefore recently added to our protocol [^11^C]palmitate PET to measure cardiac and liver NEFA fractional uptake, oxidation, and esterification rates (and liver VLDL fatty acid secretion rate) (72–74). Using the sequential intravenous [^11^C]palmitate and orally administered [^18^F]FTHA PET methods with orally administered and intravenous stable isotope palmitate tracers, we established a method to directly measure cardiac and hepatic NEFA + DFA flux over the entire 6-h postprandial period (60). The critical data from intravenous [^11^C]palmitate PET are the organ’s palmitate fractional uptake kinetic constants (K1), calculated from multicompartmental modeling of dynamic PET acquisition. In pigs, K1 does not vary markedly despite variations in blood insulin levels (72). In humans, in vivo hepatic and cardiac palmitate uptake rates increase linearly with respect to plasma palmitate levels across a wide range of plasma palmitate concentrations (75). The invariable palmitate fractional uptake in the heart and liver as a function of plasma NEFA and insulin levels suggests that it is possible to extrapolate [^11^C]palmitate K1 to the entire postprandial period in these organs. Knowing the fraction of plasma NEFA appearance that originates from intracellular WAT versus DFA spillover throughout the 6-h postprandial period from intravenous and oral administration of ^13^C and ^2^H-labeled palmitate tracers allows for the calculation of the entire first 6-h postprandial uptake of NEFA in the heart and liver in humans.

**FIGURE 2.**
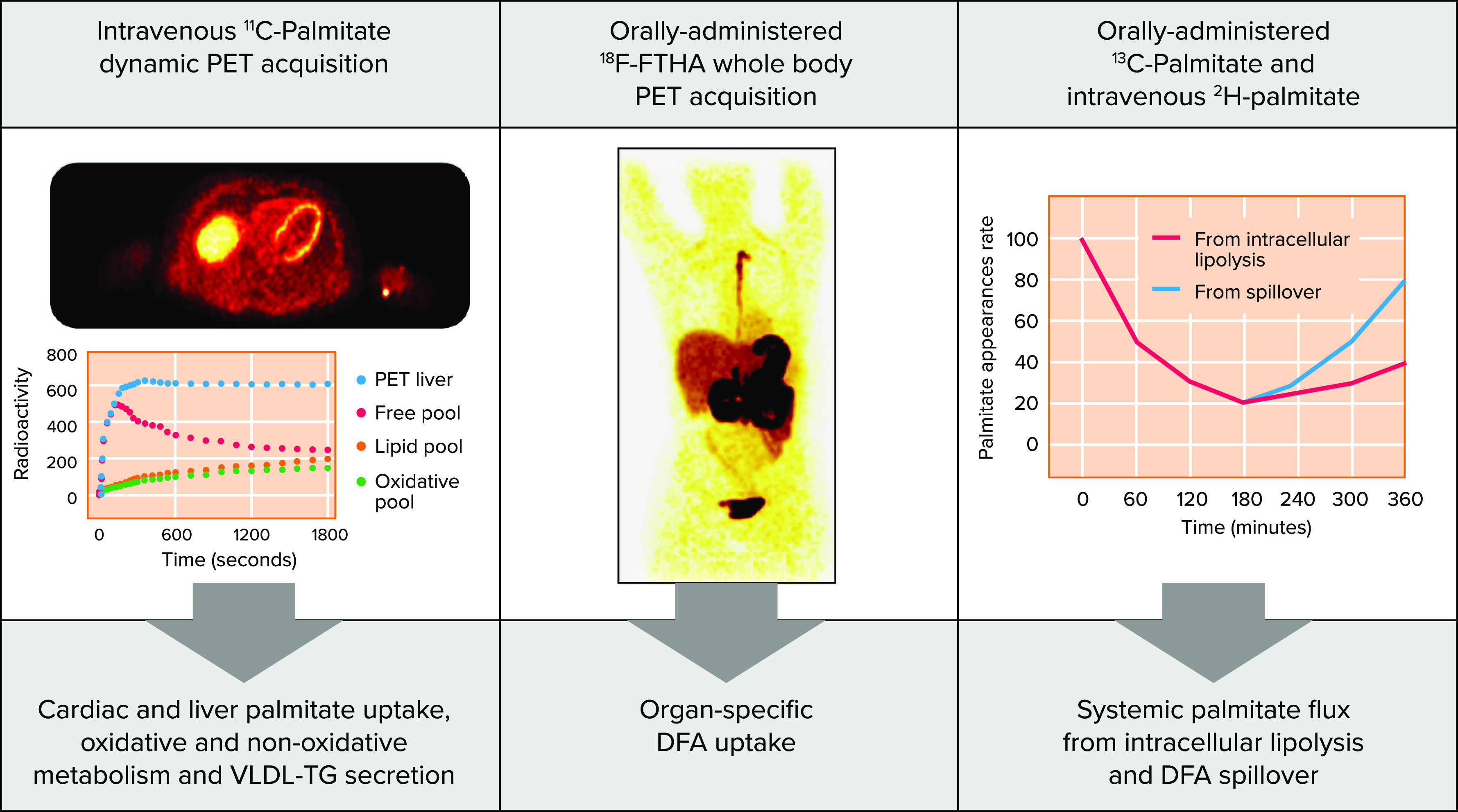
**Sequential intravenous [^11^C]palmitate and orally administered [^18^F]fluoro-thia-heptadecanoic acid positron emission tomography with orally administered and intravenous stable isotopic palmitate tracers to measure total postprandial fatty acid flux to the liver and the heart** ^818^FTHA, [^18^F]fluoro-thia-heptadecanoic acid; DFA, dietary fatty acid; PET, positron emission tomography; TG, triglycerides; VLDL, very-low density lipoprotein.

Orally administered [^18^F]FTHA, in turn, allows for the calculation of the organ’s DFA partitioning over the 6-h postprandial period (60). Correction for the gastrointestinal DFA retention [using a deep-learning graphical interface that we developed for computed tomography images semantic segmentation (76)] and knowledge of the quantity of DFA administered during the test meal then allows the calculation of 6-h postprandial uptake of DFA in organs. In the liver, this integrated 6-h postprandial DFA uptake can be further corrected for the DFA released into VLDL-TG from the hepatic [^11^C]palmitate multicompartmental modeling and systemic DFA spillover rate (60). Using these tracer and PET imaging methods sequentially in the same subjects (FIGURE 2), we found that DFA contributes 25 to 30% of total hepatic fatty acid uptake over 6 hours after meals (42, 60), in line with estimations from indirect methods using VLDL-TG tracer enrichment (31, 37, 74). In subjects living with prediabetes, WAT DFA uptake, especially in intra-abdominal fat depots, is increased while DFA flux to the liver is reduced (60, 67, 74). This increased WAT DFA partitioning occurs in the face of higher postprandial NEFA flux from WAT intracellular lipolysis and higher total postprandial fatty acid (i.e., NEFA + DFA) flux to the liver in subjects living with prediabetes (60, 67, 74).

Taken together, the results from our postprandial investigations using intravenous and oral tracers and PET imaging show a very dynamic adaptation of WAT DFA uptake and trapping upon change in lifestyle or bariatric surgery. The magnitude and even direction of these organ-specific metabolic changes could not always be predicted a priori, demonstrating the limits of our current understanding of postprandial fatty acid metabolic adaptations in pathophysiological situations. Our results currently suggest that peripheral and intra-abdominal WAT take up DFA to limit lean organ exposure to potentially toxic DFA. This adaptive mechanism is still present in subjects with obesity and T2D and can be very rapidly stimulated with interventions, such as after bariatric surgery. Whether this mechanism plays a role in the very rapid improvement in glucose metabolism occurring in T2D after bariatric surgery is the subject of current investigations.

## Investigation of BAT Metabolism in Humans Using Combination of Stable Isotope Tracers and Positron Emission Tomography

The rediscovery of functional BAT in human adults with the use of [^18^F]fluorodeoxyglucose ([^18^F]FDG) PET (77–81) has transformed the view of the pathophysiological role of adipose tissues in energy balance. Metabolically activated by cold exposure through a central nervous system sympathetic signal, these [^18^F]FDG-positive fat depots, mostly located in the supraclavicular regions in adult humans (82), display enhanced oxidative metabolism compared to other adipose tissue depots (83–85). In addition to these “classical” BAT depots, browning of WAT can also occur. From a primarily fat storage organ, adipose tissues are now also regarded as a bona fide thermogenic system, thus playing an additional role in energy substrate oxidation. Current knowledge gathered in large part from tracer and molecular imaging studies indicates that the primary energy source of BAT for thermogenesis is fatty acids from lipolysis of its own TG content [see our detailed recent review (86)]. As intracellular TG lipolysis and fatty acid oxidation occur, uptake of circulating glucose and amino acids is stimulated and drives de novo lipogenesis, contributing to replenishing BAT intracellular TG. BAT uptake of circulating NEFA is also activated during cold exposure (87, 88), contributing to enhanced fatty acid oxidation and esterification in this tissue. This activation of TG/fatty acid cycling also simultaneously occurs in WAT upon sympathetic stimulation and potentially contributes to a significant fraction of energy expenditure during acute cold exposure through futile energy substrate cycling (89). In rodents, WAT browning upon chronic cold exposure is not associated with increased in situ substrate oxidation (90), but it is characterized by more rapid TG/fatty acid cycling (91). In vivo, the cold-induced metabolic response of BAT and WAT thus constitutes an integrated thermogenic unit.

**FIGURE 3.**
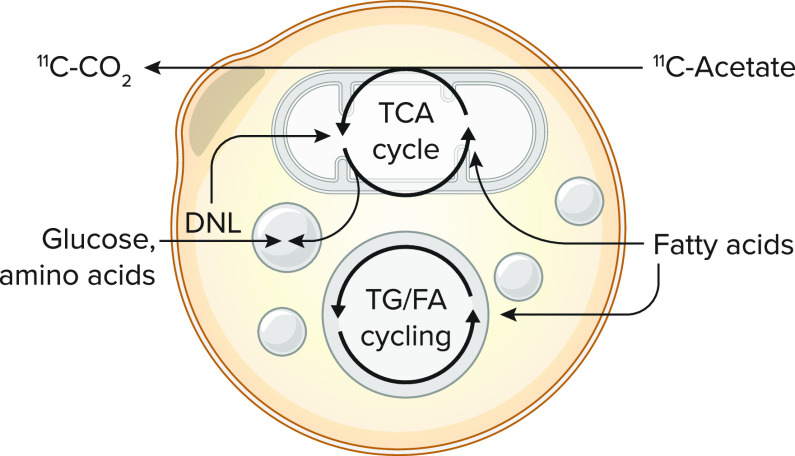
The use of [^11^C]acetate to measure brown adipose tissue oxidative metabolism DNL, de novo lipogenesis; FA, fatty acids; TCA, tricarboxylic acid; TG, triglycerides.

We have introduced [^11^C]acetate PET dynamic scanning in the field of BAT investigation in humans (83) (FIGURE 3). This tracer was initially used to determine myocardial blood flow and oxygen consumption (92). The first pass tissue uptake of [^11^C]acetate is proportional to blood flow (93, 94), whereas its rapid disappearance rate from tissues reflects [^11^C]CO_2_ back diffusion from [^11^C]acetyl-CoA oxidation in the tricarboxylic acid cycle (95). We initially used a simple mono-exponential decay modeling of BAT [^11^C]acetate time-activity curve as a semiquantitative measure of BAT oxidative metabolism (83). However, multicompartmental modeling now offers the potential to assess nonoxidative [^11^C]acetate metabolism in BAT (96) by estimating the rate of TG droplet repletion from de novo lipogenesis (92, 97).

Using this PET dynamic scanning method combined with a cooling suit system for accurate and reproducible cold exposure, we made the first demonstration of BAT cold-induced thermogenic activity in humans (83), which has been confirmed shortly after by the use of [^15^O]O_2_ PET scanning (84, 85). We furthermore were able to show that 4 wk of cold acclimation can increase this acute cold-induced BAT thermogenic response in healthy men (98). One major limitation of these current PET-based measurements is the incapacity to measure the thermogenic response of all of the anatomical BAT depots that are distributed widely from the neck to the abdomen because of the limited field-of-view of current PET scanners combined with the need to continuously capture the very rapid tissue kinetics of [^11^C]acetate and [^15^O]O_2_. Studies have thus far used PET dynamic scanning of the supra-clavicular regions with [^11^C]acetate or [^15^O]O_2_ to determine BAT oxidative activity and [^18^F]FDG PET-determined BAT total volume to extrapolate total BAT thermogenic capacity (86). The sequential use of [^11^C]acetate and [^18^F]FDG PET dynamic acquisitions demonstrated a major reduction in cold-induced BAT glucose uptake despite a similar thermogenic response of supraclavicular BAT depots in older subjects without and with T2D versus young healthy subjects (87). Cold-induced BAT [^18^F]FDG uptake can also be rapidly reduced by high fructose feeding without a change in BAT thermogenic activity in healthy men (50). Thus BAT volume determined using [^18^F]FDG PET is influenced by other factors than thermogenic activity per se and will not necessarily capture thermogenic activity nor the full volume of thermogenically active adipose tissues. This volume may potentially be up to 2.5 kg in some individuals according to CT mapping of thermochemically active adipose depots (99), as opposed to the 50- to 200-g range according to [^18^F]FDG PET (100).

Typically, acute mild cold exposure leads to a ∼1.5-fold increase in resting energy expenditure (86). BAT thermogenesis could potentially be estimated by cold-induced total body thermogenesis using personal cooling methods to minimize muscle shivering and thermogenic activity (101). However, even minimizing shivering with such methods is associated with increased deep-muscle metabolic activity that may significantly contribute to cold-induced whole body energy expenditure (102). Furthermore, increased TG/fatty acid cycling that occurs in WAT upon cold exposure may also significantly contribute to energy expenditure (89). Finally, cardiac, hepatic, and renal metabolic responses to cold may also contribute to whole body energy expenditure, although these contributions have not yet been specifically quantified in this condition.

The combination of conventional intravenous glucose tracers and [^18^F]FDG PET has allowed the direct measurement of the contribution of BAT to systemic glucose clearance (83, 102). During acute, mild cold exposure, this contribution is very small: ∼1% of systemic glucose clearance, or a few grams of glucose extrapolated over a 24-h period (100). We therefore can confidently exclude a significant role for acute cold-induced activation of BAT as a therapeutic target to improve systemic glucose clearance in humans (86). Because of the current limitations of thermogenically active BAT volume discussed above, we however cannot currently exclude some contribution of BAT to chronic energy expenditure, which ranges from insignificant (a few kcal/day) to clinically significant (ten to hundreds kcal/day) by current estimations (100). Studies using large field-of-view dynamic [^15^O]O_2_ or [^11^C]acetate PET including all BAT depots from the neck, thorax, and abdomen should be able to better quantify the full thermogenic potential of BAT depots in humans.

## Knowledge Gaps and Perspectives for Future Investigations

One important gap of in vivo lipid pathophysiological investigation is our limited capacity to specifically measure de novo lipogenesis and fatty acid esterification, which are important lipotoxic processes at the organ level. While [^11^C]acetate (92, 97) and [^11^C]palmitate (72–74) PET dynamic scanning can derive estimates of these rates, these methods assume that the slow tissue retention rate of these tracers is entirely from lipogenesis and esterification, respectively. In vivo ^2^H magnetic resonance spectroscopy (i.e., deuterium imaging) using ^2^H-labeled water and fatty acids has the potential to directly address this question using high-field magnetic resonance scanners given the very low natural tissue abundance of ^2^H and capacity to measure ^2^H enrichment specifically in tissue TG. In vivo measurement of tissue glucose metabolism has been validated using such a method with ^2^H-labeled glucose (103) and has been used to study BAT glucose metabolism in rodents (104). The combined use of [^11^C]palmitate PET and ^2^H-palmitate deuterium imaging has the potential to directly quantify tissue TG/fatty acid cycling rate in BAT and in WAT.

Our knowledge regarding organ-specific fatty acid metabolism in vivo in humans was generated mostly from [^11^C]palmitate or [^18^F]FTHA PET studies. Palmitate (the main saturated fatty acid) induces more inflammation, insulin resistance, and beta-cell dysfunction (105, 106), is higher in circulation (67), and accumulates more at the whole body level in insulin-resistant subjects than oleate and linoleate (the main mono and polyunsaturated fatty acids) (107). Thus, tissue metabolism of these fatty acids likely differs under various pathophysiological conditions. To our knowledge, there has been no direct organ-specific measurement of mono and polyunsaturated fatty acid metabolism for comparison with palmitate in vivo in humans. Sequential PET acquisitions with ^11^C-labeled fatty acid tracers should address this gap.

Recent advances in metabolomics and lipidomics make stable isotope tracer studies even more informative and accelerate the discovery process by allowing simultaneous and unbiased measurement of broad-ranging metabolic pathways in blood and organs. A recent example is the systematic metabolic profiling of blood and organs in mice after oral gavage with [U-^13^C]-glucose, which revealed a potential contribution of BAT de novo lipogenesis to circulating palmitate utilization by other organs such as the heart (108). An inherent difficulty of metabolomics approaches is their incapacity to fully quantify metabolic fluxes (i.e., organ uptake rates and tissue production of ^13^CO_2_). Combining such stable isotope tracer-based metabolomics and lipodomics approaches with PET or MRI-based imaging methods has the potential to fully and quantitatively capture the organ-specific oxidative and nonoxidative metabolic pathways of specific dietary metabolites in animal models.

These tracer and molecular imaging tools applicable directly in humans are ideal for monitoring the mechanisms leading to tissue fatty acid overexposure and lipotoxicity and driving BAT thermogenesis. Our investigations of organ-specific changes in fatty acid metabolism in the setting of prediabetes and T2D after lifestyle interventions or bariatric surgery suggest that these metabolic adaptations, overlooked until recently, are very rapid and may constitute new early targets for the monitoring of progression or remission of cardiometabolic diseases.
